# Retraction: MicroRNA-330 Is an Oncogenic Factor in Glioblastoma Cells by Regulating SH3GL2 Gene

**DOI:** 10.1371/journal.pone.0297950

**Published:** 2024-01-24

**Authors:** The PLOS ONE Editors

After this article [1] was published, concerns were raised about results reported in Figs 3 and 5.

Specifically:

- In Fig 3B:
  ○ There appear to be horizontal discontinuities across the length of both panels between the rows of bands.
  ○ There appear to be vertical discontinuities between lanes 1 and 2, and between lanes 4 and 5 in the top row of the U87 panel.
  ○ When levels are adjusted to visualize the background, the area around the band in lane 5 in the top row of the U87 panel appears to be discontinuous with the surrounding area.
- In Fig 5A:
  ○ The bottom section of the Mock panel appears similar to the top section of the Pre-miR-con panel with some different features in the top left of the overlapping section.
  ○ The bottom left of the Pre-miR-con panel appears similar to the top right of the Anti-miR-con panel.
  ○ Similarities were noted between two regions in the central section of the Anti-miR-330 panel.
- The following panels in Fig 5 in [1] appear similar to the following panels in [2] when rotated 180°:
  ○ The Mock panel in Fig 5A in [1] and the EC mimic control panel in Fig 3D in [2].
  ○ The Pre-miR-con panel in Fig 5A in [1] and the EC inhibitor control panel in Fig 3D in [2].
  ○ The Pre-miR-330 panel in Fig 5A in [1] and the EC miR-7a inhibitor panel in Fig 3D in [2].
  ○ The Anti-miR-330 panel in Fig 5A and the EC miR-7a mimic control panel in Fig 3D in [2].
  ○ The Mock panel in Fig 5B in [1] and the RP mimic control panel in Fig 3D in [2].
  ○ The Pre-miR-330 panel in Fig 5B in [1] and the RP miR-7a inhibitor panel in Fig 3D in [2].
  ○ The Anti-miR-con panel in Fig 5B in [1] and the RP inhibitor control panel in Fig 3D in [2].
  ○ The Anti-miR-330 panel in Fig 5B in [1] and the RP miR-7a mimic panel in Fig 3D in [2].
  ○ The Mock panel in Fig 5D in [1] and the far left panel in Fig 5E in [2].
  ○ The Pre-miR-con panel in Fig 5D in [1] and the far right panel in Fig 5E in [2].
  ○ The Pre-miR-330 panel in Fig 5D in [1] and the second left panel in Fig 5E in [2].
  ○ The Anti-miR-330 panel in Fig 5D in [1] and the second right panel in Fig 5E in [2].

The authors did not respond to queries about the experiments in Figs 3 and 5.

During editorial follow up, it came to light that one of the cell lines used in this article [1], U87, described as a human glioblastoma cell line in the article [1], has subsequently been identified as a contaminated cell line, and has been shown to be a glioblastoma cell line of unknown origin [3].

In light of the unresolved concerns with Figs 3B and 5A discussed above, the *PLOS ONE* Editors retract this article.

All authors either did not respond directly or could not be reached.
